# Synthesis, Characterization and Biological Evaluation of Succinate Prodrugs of Curcuminoids for Colon Cancer Treatment

**DOI:** 10.3390/molecules16021888

**Published:** 2011-02-22

**Authors:** Wisut Wichitnithad, Ubonthip Nimmannit, Sumrit Wacharasindhu, Pornchai Rojsitthisak

**Affiliations:** 1 Pharmaceutical Technology (International) Program, Faculty of Pharmaceutical Sciences, Chulalongkorn University, Bangkok 10330, Thailand; E-Mail: wisutrd@yahoo.com; 2 National Nanotechnology Center (Nanotec), National Science and Technology Development Agency, Pathumthani 12120, Thailand; E-Mail: ubonthip.n@chula.ac.th; 3 Natural Products Research Unit, Department of Chemistry, Faculty of Sciences, Chulalongkorn University, Bangkok 10330, Thailand; E-Mail: sumrit.w@chula.ac.th; 4 Department of Food and Pharmaceutical Chemistry, Faculty of Pharmaceutical Sciences, Chulalongkorn University, Bangkok 10330, Thailand

**Keywords:** curcumin, prodrug, succinylation, stability, hydrolysis

## Abstract

A novel series of succinyl derivatives of three curcuminoids were synthesized as potential prodrugs. Symmetrical (curcumin and bisdesmethoxycurcumin) and unsymmetrical (desmethoxycurcumin) curcuminoids were prepared through aldol condensation of 2,4-pentanedione with different benzaldehydes. Esterification of these compounds with a methyl or ethyl ester of succinyl chloride gave the corresponding succinate prodrugs in excellent yields. Anticolon cancer activity of the compounds was evaluated using Caco-2 cells. The succinate prodrugs had IC_50_ values in the 1.8–9.6 μM range, compared to IC_50_ values of 3.3–4.9 μM for the parent compounds. Curcumin diethyl disuccinate exhibited the highest potency and was chosen for stability studies. Hydrolysis of this compound in phosphate buffer at pH 7.4 and in human plasma followed pseudo first-order kinetics. In phosphate buffer, the *k_obs_* and *t_1/2_* for hydrolysis indicated that the compound was much more stable than curcumin. In human plasma, this compound was able to release curcumin, therefore our results suggest that succinate prodrugs of curcuminoids are stable in phosphate buffer, release the parent curcumin derivatives readily in human plasma, and show anti-colon cancer activity.

## 1. Introduction 

**Figure 1:**
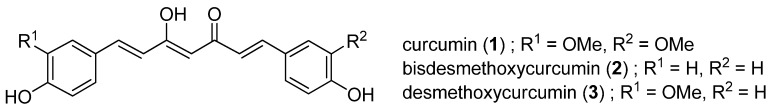
Curcuminoid structures.

Curcumin (**1)**, bisdesmethoxycurcumin (**2)** and desmethoxycurcumin (**3)** (Figure 1) are well known curcuminoids that are found in turmeric (*Curcuma longa* L., Zingiberaceae). Curcuminoids are dietary polyphenolic compounds that are of interest from the use of turmeric plants as herbal medicines. Since their initial isolation in 1870 [1], the pharmacological activities of curcuminoids have been widely investigated, and the antioxidative, anti-inflammatory, and antimicrobial properties of these molecules are well established [2,3,4,5,6,7]. Evidence has also been found for antiproliferative properties [8], an inhibitory effect against HIV-I integrase as a promising HIV treatment strategy [9], and a cholesterol-lowering effect [10]. The most attractive feature of curcuminoids is the lack of significant toxicity, as shown in several animal and human studies [11,12,13,14]. Thus, the various pharmacological effects and excellent safety profile make these molecules of interest as lead compounds for treatment of human diseases. 

The major obstacle in clinical trials has been the low bioavailability of curcuminoids due to their instability in a biological environment, and inadequate absorption and fast metabolism resulting in rapid systematic elimination. These drawbacks require administration at a high dose to achieve a significant intracellular concentration [15]. Phenolic groups and the 1,3-dicarbonyl moiety are the sites of *in vitro* and *in vivo* degradation through pathways including oxidation and hydrolysis [16]. Many strategies have been used to enhance the bioavailability of curcumin. Protection of ionization of phenolic groups and elimination of electron delocalization on the structure have been shown to improve curcuminoid stability and result in increased bioavailability. This has been accomplished by conjugation of small endogenous molecules such as amino acids, acetic acid, glucose and nucleic acids at the phenolic groups [6,17,18]. Succinic acid derivatives may also be useful in this approach, but conjugation of these molecules with curcuminoids has not been investigated. 

Attachment of a succinic acid derivative to a drug molecule (succinylation) is usually carried out by formation of an ester bond between a hydroxyl group of the target molecule and one of the carboxylic acids of the succinic acid. Since succinic acid contains two carboxylic acids, the remaining free carboxylic group can cause autocatalysis that results in instability of the succinyl products. Therefore, the free carboxylic acid is usually protected by esterification with a simple alcohol (a succinic acid monoester derivative). Succinic acid derivatives are attached to target drugs to produce more desirable physical and biological properties, with the goal that the resulting conjugates are degraded by cellular enzymes to release the target drugs *in vivo*. Therefore, succinylation falls into the prodrug category, and has the advantage of safety because succinic acid, the by-product after cleavage of the conjugate, is an endogenous substance. Succinylation has been carried out successfully for several drugs on the market, such as methylprednisolone [19], hydrocortisone [20], chloramphenicol [21], metronidazole [22] and primaquine [23]. Therefore, succinylation is suitable for development of therapeutic agents, including those based on phenolic compounds such as curcuminoids.

**Figure 2:**
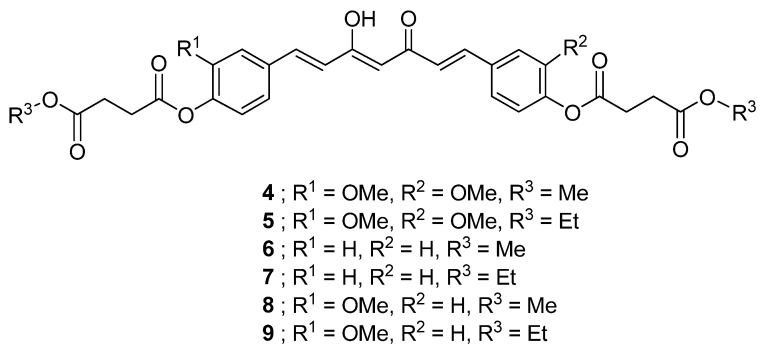
Structures of succinate prodrugs of curcuminoids.

Herein, we report the synthesis of six novel curcuminoid prodrugs **4**-**9** (Figure 2) carrying succinyl ester moieties with enhanced stability and anticolon cancer activity. The anticancer activity of compounds **1**-**9** was evaluated in colon cancer cell lines. Curcumin diethyl disuccinate (**5**) that exhibited the highest potency was chosen for further investigation of stability in phosphate buffer and curcumin release in human plasma. 

## 2. Results and Discussion

### 2.1. Synthesis of Curcuminoids ***1-3*** and Succinyl Derivatives ***4-9***

**Scheme 1:**
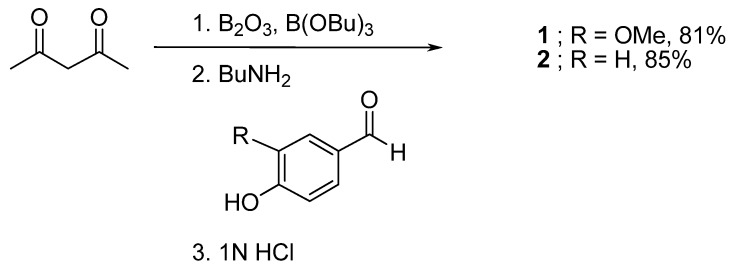
Syntheses of curcumin (**1**) and bisdesmethoxycurcumin (**2**).

Symmetrical curcuminoids [curcumin (**1**) and bisdesmethoxycurcumin (**2**)] were synthesized according to the Pabon method, as described in Scheme 1 [24]. To prevent Knovenagel condensation at the C-3 methylene group, boric anhydride was first reacted with acetylacetone to generate an acetone-boric oxide complex. The less active methyl moieties were then reacted further with an appropriate ratio of vanillin (3-methoxy-4-hydroxybenzaldehyde) to ensure double aldol condensation at both ends of the 2,4-pentanedione moiety. The reaction was performed in ethyl acetate, which provided the proper solubility for both reactants and intermediates and can be easily removed from the product, resulting in low contamination with solvent. Because the reaction is water-sensitive, tributyl borate was used as a desiccant. Tributyl borate also increases the partial positive charge at the carbonyl carbon of vanillin, resulting in an increased reaction rate. After decomplexation with 1N hydrochloric acid, compound **1** was obtained in excellent yield. NMR and other data support a curcumin purity of >99% after recrystallization in methanol. Similarly, reaction of the acetyl acetone-boric oxide complex with 4-hydroxybenzaldehyde produced **2** in excellent yield and with high purity.

**Scheme 2:**
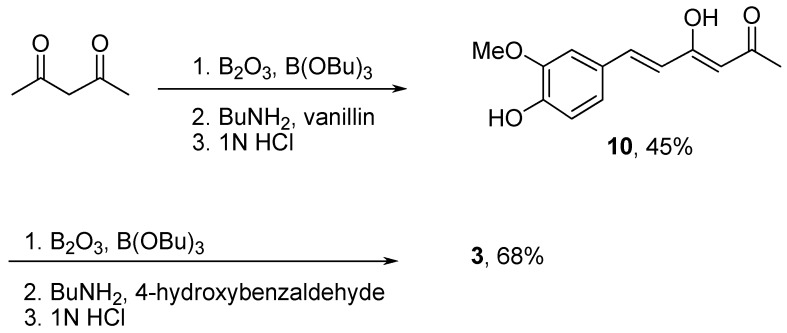
Synthesis of desmethoxycurcumin (**3**).

A synthetic method for the asymmetrical curcuminoid, desmethoxycurcumin (**3**), is presented in Scheme 2. The monosubstituted intermediate **10** were first prepared by reacting vanillin with acetyl acetone. Subsequently, **10** was subjected to a second aldol condensation with 4-hydroxybenzaldehyde to give **3** after column chromatography. The synthesized curcuminoids were fully characterized by IR, NMR and MS and the results were in agreement with previous data [25,26]. 

**Scheme 3:**
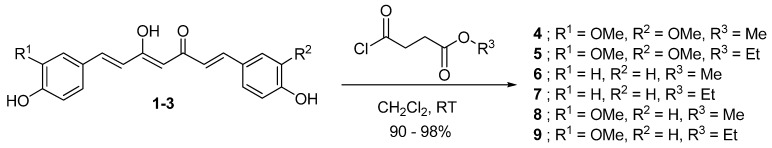
Synthesis of succinate prodrugs of curcuminoids.

With all three curcuminoids in hand, we next began synthesis of the curcuminoid-succinate prodrugs, as shown in Scheme 3. Curcumin (**1**) was first reacted with methyl-4-chloro-4-oxobutyrate or ethyl-4 chloro-4-oxobutyrate in the presence of 4-(*N,N*-dimethylamino)pyridine (DMAP) as the activator to obtain **4** and **5**, respectively. In the same manner, bisdesmethoxycurcumin (**2**) and desmethoxycurcumin (**3**) were converted into the desired prodrugs **6**-**9** upon treatment with the corresponding acid chlorides. All ester prodrugs **4**-**9** were obtained in excellent yields as yellow solids after recrystallization from methanol. The synthesized prodrugs were fully characterized by IR, NMR and MS.

### 2.2. Cytotoxicity Evaluation

The anticancer activity of curcumin-succinate prodrugs **4**–**9** against human epithelial colorectal adenocarcinoma cells (Caco-2 cells) was compared with that of the parent curcuminoids **1**–**3**. The cytotoxicity values expressed as IC_50_ values are shown in Table 1. Compounds **4–9** were all active, with IC_50_ values in the 1.8–9.6 μM range. Curcumin diethyl disuccinate (**5**) exhibited the highest potency and desmethoxycurcumin diethyl disuccinate (**9**) had the lowest potency. The other prodrugs inhibited cell growth with potency comparable to that of their parent compounds. Therefore, compound **5** was chosen for further investigation of stability in phosphate buffer and curcumin release in human plasma.

### 2.3. Chemical Stability Study

**Figure 3:**
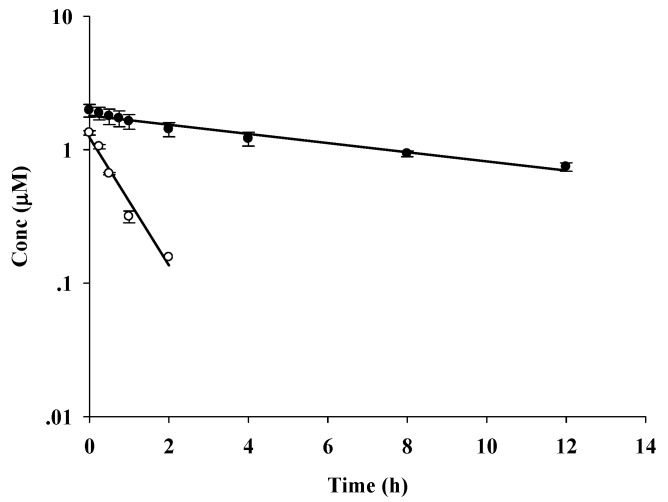
Pseudo first-order plots for degradation of curcumin (**1**; ○) and curcumin diethyl disuccinate (**5**; ●) in 0.1 M phosphate buffer (pH 7.4) at 37 °C.

The chemical stability of curcumin (**1**) and curcumin diethyl disuccinate (**5**) in 0.1 M phosphate buffer (pH 7.4) at 37 °C was determined for 12 h using reversed-phase HPLC with UV-VIS detection at 400 nm. No curcumin was found after 2 h. Semi-logarithmic plots of the concentrations of **1** and **5** in the buffer *vs.* time (Figure 3) were linear, indicating that degradation followed pseudo first-order kinetics. The overall degradation rate constants (*k_obs_*) and half-lives (*t_1/2_*) were 1.239 h^−1^ and 0.56 h, respectively, for **1** and 0.0795 h^−1^ and 7.66 h for **5**, respectively. These results indicate that succinylation significantly enhanced the chemical stability of curcumin against hydrolytic degradation at pH 7.4. This suggests that delivery of the prodrug via an intravenous route may be viable. To examine the potential oral bioavailability of the curcuminoid-succinate prodrugs, we plan to perform a chemical stability study in acidic pH conditions similar to those in the gastrointestinal tract.

We note that there are two ester groups in compound **5:** a phenolate ester and an alkoxyl (methyl or ethyl) ester. The phenolate ester is more prone to hydrolysis since phenolate anion is a better leaving group than methoxylate or ethoxylate anions. Therefore, cleavage of prodrug **5** is likely to take place mainly at the phenolate ester, which is the closer ester to the parent curcumin.

### 2.4. Release Study

**Figure 4:**
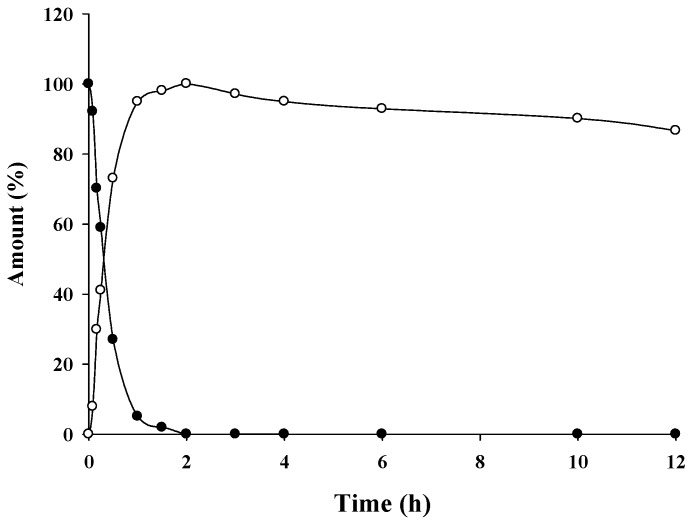
Release profiles of curcumin (**1**; ○) from curcumin diethyl disuccinate (**5**; ●) in human plasma at 37 °C.

Release of curcumin from curcumin diethyl disuccinate (**5**) in human plasma was monitored by measuring the depletion of **5** and the increase of curcumin using reversed-phase HPLC with UV-VIS detection at 400 nm. The amount of curcumin released was consistent with the decrease in **5** (Figure 4). All curcumin was released within 2 h. The release of curcuminoids may be mainly due to enzymatic hydrolysis by esterases in human plasma. The ability of **5** to release the parent curcumin suggests that succinylation could be useful as a potential prodrug approach for curcuminoids. 

## 3. Experimental

### 3.1. General

All reagents and solvents were purchased from commercial sources and used without further purification. Reactions were performed under nitrogen and monitored by TLC. Flash chromatography was performed on 230–400 mesh silica gel with technical grade solvents. ^1^H- and ^13^C-NMR spectra were recorded on a Varian Inova Fourier Transform NMR 500 MHz spectrometer operated with the VnmrJ software. NMR spectra were obtained in deuterated chloroform (CDCl_3_) or deuterated dimethylsulfoxide (DMSO). Chemical shifts are reported as δ values in parts per million (ppm) relative to the residual solvent peak and coupling constants are reported as *J* values in Hertz (Hz). IR spectra were recorded with a Perkin Elmer 1760 X. High resolution mass spectra were obtained on a Reflex IV Bruker time-of-flight High-Resolution Mass Spectrometer (HRMS). Melting points were determined using a Differential Scanning Calorimeter (DSC823^e^, Mettler Toledo). The amount of curcumin (**1**) and curcumin diethyl disuccinate (**5**) were determined using an Agilent 1200 HPLC system (Agilent, CA, USA) equipped with an Alltech Alltima C18 column (150 × 4.6 mm i.d., 5μ, Grace, IL, USA).

### 3.2. General Method for the Synthesis of Curcumin *(**1**)* and Bisdesmethoxycurcumin *(**2**)*

Acetylacetone (1.03 mL, 10 mmol) was added to a solution of boric anhydride (0.35 g, 5.0 mmol) in ethyl acetate (30 mL), followed by addition of vanillin (3.04 g, 20 mmol) or 4-hydroxybenzaldehyde (2.44 g, 20 mmol) and tributyl borate (10.8 mL, 40 mmol). The reaction mixture was stirred at 50 °C for 5 min. Subsequently, *n*-butylamine (0.4 mL, 5.0 mmol) in ethyl acetate (5 mL) was added dropwise over 15 min at 50 °C and additionally stirred for 4 hours at 80 °C. Hydrochloric acid (1 N, 30 mL) was added and the mixture was stirred for another 30 min. The organic layers were separated and extracted with ethyl acetate (3 × 30 mL). The combined organic layer was washed with water. The organic layer was dried over sodium sulfate, filtered and concentrated under reduced pressure. The crude product was recrystallized from methanol to give the corresponding curcuminoids as yellow solids.

*(1E,6E)-1,7-bis(4-Hydroxy-3-methoxyphenyl)hepta-1,6-diene-3,5-dione* (**1**, curcumin). Yield 81%; m.p. 187–188 °C (lit. 184–185 °C) [25]; IR (KBr) 3,500, 1,626, 1,601, 1,504, 1,427, 1,261, 1,026 cm^−1^; ^1^H- NMR (CDCl_3_) δ 3.95 (s, 6H), 5.80 (s, 1H) 6.48 (d, *J* = 15.7 Hz, 2H), 6.94 (d, *J* = 8.1 Hz, 2H), 7.05 (d, *J* = 1.8 Hz, 2H), 7.13 (dd, *J* = 8.1 and 1.8 Hz, 1H), 7.59 (d, *J* = 15.7 Hz, 2H); ^13^C-NMR (CDCl_3_) 183.3, 147.8, 146.8, 140.5, 127.6, 122.8, 121.8, 114.8, 109.6, 101.2, 56.0; HRMS calcd. for C_21_H_21_O_6_ [M+H^+^]: 369.1338; found 369.1335.

*(1E,6E)-1,7-bis(4-Hydroxyphenyl)hepta-1,6-diene-3,5-dione* (**2**, bisdesmethoxycurcumin). Yield 85%; m.p. 233–234 °C (lit. 231–232 °C) [25]; IR (KBr) 3,184, 1,626, 1,620, 1,509, 1,431, 1,235, 1,103 cm^−1^; ^1^H-NMR (DMSO-*d_6_*) δ 6.07 (s, 1H), 6.69 (d, *J* = 15.9 Hz, 2H), 6.85 (d, *J* = 8.6 Hz, 4H), 7.56 (d, *J* = 8.6 Hz, 4H), 7.56 (d, *J* = 15.9 Hz, 2H), 10.25 (s, 2H); ^13^C-NMR (CDCl_3_) 183.6, 160.1, 140.8, 130.7, 126.2, 121.1, 116.3, 101.4; HRMS calcd. for C_19_H_16_O_4_ [M+H^+^]: 309.1127; found 309.1125.

### 3.3. Synthesis of Desmethoxycurcumin *(**3**)*.

A complex of 2,4-pentanedione (acetylacetone) (4.12 mL, 40 mmol) with boric anhydride (0.69 g, 10 mmol) was formed in ethyl acetate (30 mL) by reaction at 80 °C for 30 min. A mixture of vanillin (1.52 g, 10 mmol) and tributyl borate (5.38 mL, 20 mmol) in dried ethyl acetate (10 mL) was then added to the acetylacetone-boron complex mixture. The solution was stirred vigorously for 15 min at 50 °C, with dropwise addition of *n*-butylamine (0.73 mL, 10 mmol) in ethyl acetate (5 mL) over 15 min. The reaction mixture gradually turned yellow. Stirring was continued overnight at room temperature. 1N HCl (30 mL) was added and the mixture was stirred for another 30 min. The organic layers were separated and the aqueous fraction was extracted with ethyl acetate (3 × 30 mL). The combined organic layer was washed with water. The organic layer was dried over anhydrous sodium sulfate as desiccant and the organic solvent was then removed under reduced pressure. After removal of the solvent under vacuum, the crude products were purified by flash column chromatography with elution with an hexane-ethyl acetate gradient to afford *4-Hydroxy-6-(4-hydroxy-3-methoxyphenyl)-hexa-3,5-dien-2-one* (furoylacetone, **10**) as a pale yellowish solid. Yield 45%; m.p. 144–146 °C (lit. 146–147 °C) [26]; IR (KBr) 3,335, 1,632, 1,561, 1,511, 1,419, 1,247, 1,029 cm^-1^; ^1^H-NMR (CDCl_3_) δ 2.14 (s, 3H), 3.91 (s, 3H), 5.61 (s, 1H), 6.30 (d, *J* = 15.8 Hz, 1H), 6.90 (d, *J* = 2.1 Hz, 1H), 7.00 (d, *J* = 1.5 Hz, 1H), 7.06 (dd, *J* = 8.2 and 1.5 Hz, 1H), 7.51 (d, *J* = 15.8 Hz, 1H); ^13^C-NMR (CDCl_3_) 196.9, 178.0, 147.7, 146.8, 140.0, 127.7, 122.6, 120.3, 114.8, 109.5, 100.6, 55.9, 26.8. A complex of furoylacetone **10** (0.40 g, 1.71 mmol) and boric anhydride (0.60 g, 0.85 mmol) was formed in ethyl acetate (15 mL). The mixture was stirred constantly at 50 °C for 30 min until the solution was milky. Subsequently, a mixture of 4-hydroxybenzaldehyde (0.21 g, 1.71 mmol) and tributyl borate (460 µL, 1.71 mmol) was added and stirred at 50 °C for 30 min. *n*-Butylamine (169 µL) in ethyl acetate (5 mL) was added dropwise over 15 min. The reaction mixture gradually turned yellowish. Stirring was continued overnight at room temperature. 1N HCl (30 mL) was added and the mixture was further stirred for 30 min. The organic layer was separated and the aqueous fraction was extracted with ethyl acetate (3 × 30 mL). The combined organic layer was washed with water. The organic layer was dried over anhydrous sodium sulfate as desiccant and the organic solvent was then removed under reduced pressure. After removal of the solvent under vacuum, the crude products were purified by flash column chromatography with elution with a hexane-ethyl acetate gradient to afford *(1E,6E)-1-(4-Hydroxy-3-methoxyphenyl)-7-(4-hydroxyphenyl)hepta-1,6-diene-3,5-dione* (desmethoxycurcumin, **3**) as a yellowish solid. Yield 68%; m.p. 174–176 °C (lit 175–176 °C) [25]; IR (KBr) 3,154, 1,630, 1,618, 1,501, 1,433, 1,225, 1,100 cm^-1^; ^1^H-NMR (CDCl_3_) δ 3.93 (s, 3H), 5.78 (s, 1H), 6.46 (d, *J* = 15.8 Hz, 2H), 6.84 (d, *J* = 8.5 Hz, 2H), 6.91 (d, *J* = 8.2 Hz, 1H), 7.03 (d, *J* = 1.5 Hz, 1H), 7.10 (dd, *J* = 8.2 and 1.5 Hz, 1H), 7.44 (d, *J* = 8.5 Hz, 2H), 7.57 (d, *J* = 15.8 Hz, 1H), 7.59 (d, *J* = 15.8 Hz, 1H); ^13^C-NMR (CDCl_3_) 183.6, 157.6, 147.9, 147.0, 146.9, 146.8, 140.6, 140.1, 129.9, 127.9, 127.7, 122.9, 121.8, 115.9, 114.8, 109.7, 101.2, 55.9; HRMS calcd. for C_20_H_18_O_5_ [M+H^+^]: 361.1052; found 361.1053.

### 3.4. General Method for Synthesis of Curcuminoid Succinate Ester Derivatives ***4–9***

A round-bottomed flask (50 mL) was charged with curcuminoids **1, 2** or **3** (0.54 mmol) and 4-(*N,N*-dimethylamino)pyridine (DMAP) (1.2 mmol), followed by addition of dichloromethane or 50% dichloromethane in diethyl ether (30 mL). The solution was slowly added to a solution of methyl-4-chloro-4-oxobutyrate (1.2 mmol) or ethyl-4-chloro-4-oxobutyrate (1.2 mmol) in dichloromethane (2 mL) under nitrogen at room temperature. After stirring for 2 h, the solvent was removed and the residue was diluted with ethyl acetate (20 mL). The reaction mixture then was filtered, washed with 1 M sodium bicarbonate (20 mL) and water (20 mL), dried over sodium sulfate, and concentrated under reduced pressure. The crude product was recrystallized from cold methanol to give the curcuminoid succinate ester derivatives **4**-**9** as pale yellowish solids.

*4,4’-((1E,6E)-3,5-Dioxohepta-1,6-diene-1,7-diyl)bis(2-methoxy-1,4-phenylene)dimethyl disuccinate* (**4**, curcumin dimethyl disuccinate). Yield 90%; m.p. 143–145 °C, IR (KBr) 2,985, 1,756, 1,732, 1,630, 1,598, 1,511, 1,415, 1,254, 1,204, 1,123, 1,029 cm^−1^; ^1^H-NMR (CDCl_3_) δ 2.76 (t, *J* = 6.8 and 7.0 Hz, 4H), 2.94 (t, *J* = 6.7 and 7.0 Hz, 4H), 3.73 (s, 6H), 3.87 (s, 6H), 5.86 (s, 1H), 6.56 (d, *J* = 15.9 Hz, 2H), 7.08 (d, *J* = 8.1 Hz, 2H) 7.09 (d, *J* = 1.8 Hz, 2H), 7.16 (dd, *J* = 8.1 and 1.8 Hz, 2H), 7.62 (d, *J* = 15.9 Hz, 2H). ^13^C-NMR (CDCl_3_) 183.1, 172.4, 170.3, 151.4, 141.2, 139.9, 133.9, 124.3, 123.3, 121.0, 111.5, 101.8, 55.9, 51.9, 28.9; HRMS calcd. for C_31_H_32_O_12_ [M+H^+^]: 597.1972; Found 597.1979. 

*4,4’-((1E,6E)-3,5-Dioxohepta-1,6-diene-1,7-diyl)bis(2-methoxy-1,4-phenylene)diethyl disuccinate* (**5**, curcumin diethyl disuccinate). Yield 95%; m.p. 95–96 °C, IR (KBr) 2,985, 1,746, 1,723, 1,633, 1,598, 1,509, 1,412, 1,252, 1,034 cm^−1^; ^1^H-NMR (CDCl_3_) δ 1.25 (t, *J* = 7.0 and 7.2, 6H), 2.72 (t, *J* = 6.9 and 7.0 Hz, 4H), 2.91 (t, *J* = 6.9 and 7.0 Hz, 4H), 3.84 (s, 6H), 4.16 (m, *J* = 7.2 Hz, 4H), 5.83 (s, 1H), 6.54 (d, *J* = 15.7 Hz, 2H), 7.05 (d, *J* = 8.3 Hz, 2H) 7.09 (d, *J* = 1.7 Hz, 2H), 7.16 (dd, *J* = 8.3 and 1.7 Hz, 2H), 7.58 (d, *J* = 15.7 Hz, 2H). ^13^C-NMR (CDCl_3_) 183.1, 171.9, 170.3, 151.3, 141.2, 139.9, 133.9, 124.3, 123.3, 121.0, 111.5, 101.8, 60.79, 55.9, 29.2, 28.9, 14.2; HRMS calcd. for C_33_H_36_O_12_[M+H^+^]: 625.2285; Found 625.2281. 

*4,4’-((1E,6E)-3,5-Dioxohepta-1,6-diene-1,7-diyl)bis(1,4-phenylene)dimethyl disuccinate* (**6**, bis-desmethoxycurcumin dimethyl disuccinate). 95% yield, m.p. 192–193 °C, IR (KBr) 2,935, 1,746, 1,734, 1,623, 1,560, 1,502, 1,420, 1,308, 1,115, 1,035 cm^−1^; ^1^H-NMR (CDCl_3_) δ 2.75 (t, *J* = 6.5 and 6.8 Hz, 4H), 2.89 (t, *J* =6.5 and 6.8 Hz, 4H), 3.72 (s, 6H), 5.82 (s, 1H), 6.55 (d, *J* = 15.8 Hz, 2H), 7.12 (d, *J* = 8.5 Hz, 4H), 7.54 (d, *J* = 8.6 Hz, 4H), 7.62 (d, *J* = 15.8 Hz, 2H). ^13^C-NMR (MHz, CDCl_3_) 183.1, 172.5, 170.6, 151.9, 139.5, 132.8, 129.2, 124.2, 122.1, 120.9, 101.8, 51.9, 29.3, 28.8; HRMS calcd. for C_29_H_28_O_10_ [M+H^+^]: 537.1760; Found 537.1764.

*4,4’-((1E,6E)-3,5-Dioxohepta-1,6-diene-1,7-diyl)bis(1,4-phenylene) diethyl disuccinate* (**7**, bis-desmethoxycurcumin diethyl disuccinate). Yield 97%; m.p. 155–158 °C, IR (KBr) 2,983, 2,940, 1,750, 1,724, 1,631, 1,567, 1,508, 1,416, 1,313, 1,131, 1,021 cm^−1^; ^1^H-NMR (CDCl_3_) δ 1.25 (t, *J* = 7.1 and 7.1, 6H), 2.70 (t, *J* = 6.4 and 6.8 Hz, 4H), 2.89 (t, *J* =6.4 and 6.8 Hz, 4H), 4.16 (m, *J* = 7.1, 4H), 5.81 (s, 1H), 6.55 (d, *J* = 15.8 Hz, 2H), 7.12 (d, *J* = 7.3 Hz, 4H), 7.54 (d, *J* = 7.4 Hz, 4H), 7.62 (d, *J* = 15.8 Hz, 2H). ^13^C-NMR (CDCl_3_) 183.1, 172.0, 170.7, 151.9, 139.5, 132.8, 129.2, 124.2, 122.1, 120.9, 101.8, 60.9, 29.4, 29.1, 14.2; HRMS calcd for C_31_H_32_O_10_ [M+H^+^]: 565.2073; Found 565.2081.

*4-((1E,6E)-7-(4-(4-Methoxy-4-oxobutanoyloxy)-3-methoxyphenyl)-3,5-dioxohepta-1,6-dienyl)phenyl methyl succinate* (**8**, desmethoxycurcumin dimethyl disuccinate). Yield 98%; m.p. 105–109 °C, IR (KBr) 2,947, 1,740, 1,734, 1,627, 1,596, 1,509, 1,410, 1,255, 1,044, 1,029 cm^−1^; ^1^H-NMR (CDCl_3_) δ 2.74 (m, 4H), 2.90 (m, 4H), 3.71 (s, 6H), 3.85 (s, 3H), 5.82 (s, 1H), 6.54 (d, *J* = 15.8 Hz, 1H), 6.55 (d, *J* = 15.8 Hz, 1H), 7.06 (d, *J* = 8.6 Hz, 2H) 7.04-7.14 (m, 3H), 7.55 (d, *J* = 8.6 Hz, 2H), 7.55 (d, *J* = 15.8 Hz, 1H), 7.56 (d, *J* = 15.8 Hz, 1H). ^13^C-NMR (CDCl_3_) 183.1, 183.0, 172.4, 170.6, 170.2, 151.9, 151.4, 141.3, 139.9, 139.5, 134.0,132.8, 129.2, 124.3, 124.2, 123.3, 122.1, 121.0, 111.5, 101.8, 55.9, 51.9, 51.8, 29.3, 29.0, 28.8; HRMS calcd. for C_30_H_30_O_11_[M+H^+^]: 567.1866; Found 567.1862. 

*4-((1E,6E)-7-(4-(4-Ethoxy-4-oxobutanoyloxy)-3-methoxyphenyl)-3,5-dioxohepta-1,6-dienyl)phenyl ethyl succinate* (**9**, desmethoxycurcumin diethyl disuccinate). Yield 96%; m.p. 95–97 °C, IR (KBr) 2,980, 2,936, 1,758, 1,735, 1,623, 1,584, 1,508, 1,420, 1,221, 1,070, 1,020 cm^−1^; ^1^H-NMR (CDCl_3_) δ 1.26 (t, *J* = 7.1 and 7.1 Hz, 6H), 2.72 (m, 4H), 2.89 (m, 4H), 3.85 (s, 3H), 4.16 (m, 4H), 5.82 (s, 1H), 6.54 (d, *J* = 15.8 Hz, 1H), 6.55 (d, *J* = 15.8 Hz, 1H), 7.06 (d, *J* = 8.6 Hz, 2H) 7.04–7.14 (m, 3H), 7.54 (d, *J* = 8.6 Hz, 2H), 7.54 (d, *J* = 15.8 Hz, 1H), 7.56 (d, *J* = 15.8 Hz, 1H). ^13^C-NMR (CDCl_3_) 183.2, 183.1, 172.0, 170.7, 170.3, 151.9, 151.4, 141.3, 139.9, 139.6, 134.0, 132.7, 129.2, 124.3, 124.2, 123.3, 122.1, 121.1, 111.5, 101.8, 60.9, 60.8, 55.9, 29.3, 29.2, 29.1, 28.9, 14.2; HRMS calcd for C_32_H_34_O_11_[M+H^+^]: 595.2179; Found 595.2188.

### 3.5. Cytotoxicity Evaluation

The anticancer activity of curcuminoids and their succinate prodrugs was evaluated in a colon cancer cell line: Caco-2 (human colon adenocarcinoma, ATCC Cat. No. HTB-37). Cytotoxicity was determined using a MTT microplate assay [27]. Stock solutions of curcuminoids or succinate prodrugs were prepared in dimethylsulfoxide (DMSO) at 1,000 µM. The solutions were serially diluted with the culture medium of the cells to obtain sample solutions at appropriate concentrations. The cells were exposed to the sample at a concentration range of 10 to 0.078 µM. The incubation time was 24 h, as used in previous studies of the cytotoxicity of curcumin prodrugs [28,29]. After incubation, the samples were removed from the cell cultures prior to performance of the MTT assay. Doxorubicin was used as a positive control and 0.5% DMSO as a negative control. Results are expressed as the concentration of compound required to kill 50% of the cells (IC_50_) in comparison with the control. Experiments were performed in triplicate.

### 3.6. Chemical Stability Study

Stock methanolic solutions of curcumin (**1**) and curcumin diethyl disuccinate (**5**) were separately prepared in 0.1 M potassium phosphate buffer (pH 7.4) and then diluted with the same buffer to give a final concentration of 1.5 μM. The solution was left to stand at 37 °C for 12 h. The amount of curcumin (**1**) or curcumin diethyl disuccinate (**5**) was determined at appropriate time intervals using a previously reported HPLC method with modifications [30]. Chromatography was performed using a gradient system with an autosampler temperature of 15 °C, a column temperature of 33 °C, a flow rate of 2.0 mL/min, and a detection wavelength of 400 nm. Gradient elution was performed with eluent A (2% v/v aqueous acetic acid) and B (acetonitrile). The elution program was optimized as follows: initial 0–4 min, isocratic elution A-B (70:30, v/v); 4–5 min, linear gradient to A-B (50:50, v/v); 5–8 min, isocratic elution A-B (50:50, v/v); 8–9 min, linear gradient to A-B (45:55, v/v); 9-10 min, isocratic elution A-B (45:55, v/v); 10–11 min, linear gradient to A-B (30:70, v/v); 11–12 min, isocratic elution A-B (30:70, v/v); 12–13 min, linear gradient to A-B (70:30, v/v); 13–15 min, isocratic elution A-B (70:30, v/v). The injection volume was 20 μL. Experiments were performed in triplicate. Kinetic parameters were determined by a semi-logarithmic plot of concentration vs. time and calculated using a linear least-squares regression analysis.

### 3.7. Release Study

Plasma was spiked with a stock aqueous solution of curcumin diethyl disuccinate (**5**) to give a final concentration of 1.5 μM. The spiked plasma was left to stand at 37 °C for 12 h. The release profile of curcumin was determined at appropriate time intervals by extraction with acetonitrile and analysis using the HPLC conditions described above. Experiments were performed in triplicate. 

## 4. Conclusions

In conclusion, curcuminoid-succinate prodrugs **4**–**9** were prepared via succinylation of the parent curcuminoids **1**–**3**. The six derivatives were readily synthesized in high purity and good yields. A hydrolytic study indicated improved stability of the prodrug in comparison with the parent drug. The observation of significantly increased curcumin release in human plasma suggests that succinylation of curcuminoids is an effective prodrug strategy. Therefore, prodrugs **4**–**9** may be promising candidates for future treatment of colon cancer.

## Figures and Tables

**Table 1:** *In vitro* cytotoxicity of curcuminoids **1-3** and curcuminoid-succinate prodrugs **4-9** against human epithelial colorectal adenocarcinoma (Caco-2) cells (*n* = 3).

Compounds	IC_50_ (μM) ± SD
**1**	3.31 ± 0.16
**2**	4.93 ± 0.21
**3**	3.36 ± 0.26
**4**	3.84 ± 0.19
**5**	1.84 ± 0.11
**6**	3.78 ± 0.31
**7**	5.97 ± 0.28
**8**	4.40 ± 0.15
**9**	9.60 ± 0.31
